# A Perspective on Reversibility of Tendinosis-Induced Multi-Level Adaptations

**DOI:** 10.3389/fphys.2020.00651

**Published:** 2020-07-07

**Authors:** Kornelia Kulig, Yu-Jen Chang, David Ortiz-Weissberg

**Affiliations:** ^1^Jacquelin Perry Musculoskeletal Biomechanics Research Laboratory, Division of Biokinesiology and Physical Therapy, Herman Ostrow School of Dentistry, University of Southern California, Los Angeles, CA, United States; ^2^Division of Physical Therapy, School of Medicine, West Virginia University, Morgantown, WV, United States

**Keywords:** tendinopathy, motor control, stiffness, feedforward, neuromechanics, spinal reflex, plasticity

## Abstract

Achilles tendinopathy is a well-known pathology that can display interindividual variations in chronicity, symptom presentation, and tendon morphology. Furthermore, symptoms may fluctuate within an individual throughout the stages of the pathology. Although pain is often used as a marker of condition severity, individuals may not consistently report pain due to periods of remission. Persons with tendinosis, which is characterized by advanced morphological alterations, have shown consistent changes in neuromechanics that indicate adaptations in the sensory-motor and the central nervous systems. The current treatment strategy involves repetitive resistance exercise aiming to achieve recovery of lost function. This treatment approach, however, has gauged such functional recovery through symptom relief and return to sport, which, in our opinion, may not suffice and may not prevent symptom recurrence or tendon rupture. In this physiologically informed perspective, we briefly review what is currently known about the consequences of Achilles tendon degeneration and examine the topic of reversing these changes. Shortcomings of contemporary treatment strategies are discussed and we therefore call for a new paradigm to focus on the whole-body level, targeting not only the tendon but also the reversal of the neuromotor control system adaptations.

## Introduction

Achilles tendinosis, a subcategory of Achilles tendinopathy characterized by advanced degeneration, continues to lack interventions capable of consistently reversing this degeneration and its multi-system consequences, despite the significant attention garnered in the literature. The absence of consensus on a term for capturing the combination of advanced degeneration and potentially intermittent pain (Cook et al., 2016; Scott et al., 2019) has led us to adopt the use of tendinosis, which we operationally define as imaging-quantified, increased focal tendon thickness and intratendinous disorganization with past or current tendon pain.

Our framework espouses the earlier views that the tendinopathic process begins with paratenonitis and may lead to tendinosis (Hess et al., 1989; Kannus and Józsa, 1991). Marked changes due to experimentally-induced, short-term tendinosis in rabbits were more frequent in the paratenon than in the tendon proper (Backman et al., 1990). In addition, such marked tendon changes rarely occurred without concurrent alterations in the paratenon. Similarly, in humans, a greater proportion of paratenon abnormalities in Achilles tendinopathy were accompanied by intratendinous changes as compared to intratendinous abnormalities showing concomitant paratenon changes (Karjalainen et al., 2000). This pattern continues to hold true in the case of ruptured Achilles tendons in humans: early histopathological evidence included degeneration in the tendon proper with no signs of inflammatory infiltrates (Kannus and Józsa, 1991). With regards to connections between the various processes, recent findings that inflammatory mediators seemed to stimulate the preliminary changes of tendinopathy further imply that the processes of inflammation and degeneration are not discontinuous (Behzad et al., 2013). As such, we view Achilles tendinopathy as a continuum in which the initial stage is inflammation-and-pain-dominated paratenonitis while the degenerative tendinosis stage occurs if the offending stimulus is not attenuated.

It is important to note that pain appears to follow a stimulus-response relationship and is not always reported, complicating the detection of degeneration (Maffulli et al., 1998, 2003). This is exemplified by instances where imaging of the Achilles upon rupture or first indication of pain has revealed advanced degeneration, indicating the degeneration must have begun in the absence of pain (Maffulli et al., 2003; Cook and Purdam, 2008). However, current treatment strategies often focus on pain, which can be improved in as little as 2 weeks (Murphy et al., 2018), and do not integrate recently discovered multi-level adaptations that occur in the wake of degeneration (Chang and Kulig, 2015). Therefore, there is a need to determine whether these treatment strategies are capable of reversing Achilles degeneration and its associated sequelae.

The aim of this perspective is to examine the topics of Achilles tendon degeneration, reversal of said degeneration, and the determination of whether a new treatment approach for Achilles tendinosis is warranted. From the whole-body perspective, we speculate that pain may leave a footprint on movement strategies, especially those of a repetitive nature, such as running, jumping, or cutting. Only during the pain-free period can the participant tolerate loads that stimulate material and mechanical property enhancement, giving the tendon the chance to remodel. A comprehensive treatment protocol will address not only the tendon tissue properties, but will also modify movement strategies. This statement comes with the qualifiers that (1) improvement of tendon tissue properties must precede the movement strategy modifications and (2) each portion of the treatment plan avoids inducing levels of pain that may interfere with the desired mechanical loading.

## Morphological Considerations in Achilles Tendinosis

The degenerative changes of the Achilles tendon occur on both the intratendinous (micromorphological) and the whole-tendon (macromorphological) scales, with the latter category typically referring to non-uniform changes in tendon thickness and cross-sectional area (Cook and Purdam, 2008; Kulig et al., 2013, 2014). In contrast, micromorphological alterations are much more extensive but are not always detected due to a lack of commonplace assessment outside the research setting. Sonography (with macro and micromorphology analysis) is our recommended modality as it allows for ease of access in the clinical setting and also permits analysis of the intratendinous structural integrity using recent techniques (Bashford et al., 2008; Van Schie et al., 2010).

The numerous micromorphological changes of tendinosis include decreased collagen density; disruption of collagen orientation and organization; increased sonographic hypoechogenicity; angiogenesis; and possible ingrowth of nerves (Riley, 2004; Cook and Purdam, 2008; Xu and Murrell, 2008; Klatte-Schulz et al., 2018). Although the tendinotic Achilles may show these changes at the myotendinous junction or insertion, they are most common in its mid-substance, where tendon thickening is localized (focal thickening). Ultrasound-based analyses indicated that increased focal thickness in the presence of degeneration, unlike homogenous thickness, involves separation of collagen bundles (Bashford et al., 2008; Kulig et al., 2016), which was associated with alteration of tendon material and mechanical properties. This increased distance between axially aligned collagen bundles within a given region of focal thickness was recently correlated with lower tendon modulus of elasticity and stiffness (increased compliance) (Kulig et al., 2016), providing evidence for the morphological underpinnings of tendon material and mechanical properties.

## Multi-Level Physiological Adaptations to Tendinosis

The functional consequences of long-term Achilles tendinosis extend outside the tendon to the muscle and nervous systems (Arya and Kulig, 2010; Chang and Kulig, 2015). The following adaptations were revealed through studies from our lab on subjects who had previously experienced various levels of Achilles tendon symptoms but were pain-free at the time of participation in our studies. At the tendon level, degeneration affects material and mechanical characteristics. Reductions in these properties then lower the tendon’s resistance to strain and elongation. One manifestation of this tendon compliance is increased electromechanical delay (EMD) of the triceps surae, representing the lag between muscle activation and measurable force production (Chang and Kulig, 2015). The nervous system then attempts to compensate for this temporal inefficiency by upregulating the feedforward drive, observed as earlier pre-activation of the gastrocnemius prior to the point of initial ground contact during hopping.

In addition to the supraspinal involvement implied by the aforementioned feedforward signal, adaptations to advanced tendon degeneration are also observed at the spinal level (Chang and Kulig, 2015) in the form of feedback control mechanisms. Tibial nerve motor neurons supplying the triceps surae have displayed a higher normalized H-reflex, indicating increased alpha motor neuron excitability. The altered excitability may result in part from enhanced descending neural drive from the cortex, seen as an increase in the V-wave from the tibial nerve. The presently known adaptations conclude with depression of triceps surae and tibialis anterior electromyography (EMG) amplitude. Although this reduced recruitment of the primary plantar flexors and their antagonist may occur to shield the tendon from further load, adequate force production then demands use of the secondary plantar flexors. As a result, there is increased peroneus longus activity relative to the gastrocnemius and soleus. It is reasonable to speculate that there is also altered recruitment of the other secondary plantar flexors, keeping in mind that this is currently unconfirmed. The myriad of alterations presents the challenge of identifying intervention protocols that rectify not only the tendon’s inherent properties, but that address nervous system-based adaptations occurring at the whole-body level.

## Significance of Reversibility

The understanding of the multilevel adaptations to Achilles tendinosis can benefit from considering the human motor control perspective. In human motor control terms, the tendon is the bridge between the actuator (muscle) and the plant (i.e., the skeletal system). In the presence of Achilles tendinosis, the tendon is weakened, impacting the actuators, the sensors (proprioceptors), and the controller (nervous system). The temporal efficiency of the musculotendinous unit is compromised, stimulating the central nervous system to tune both feedforward and feedback control mechanisms as compensation for the deficit of motor output. If the tendon regains its normal function, it then becomes possible to reverse the adaptations seen in the actuator and controller. The reversal of the multilevel adaptations is therefore critical in treating persons with Achilles tendinosis, and intervention should start with the enhancement of tendon modulus of elasticity and stiffness.

## Reversibility Strategies

It is our informed hypothesis that the multilevel adaptations accompanying tendinosis (Chang and Kulig, 2015), likely developed over many years, exhibit the potential for reversal. Integrating the intervention-based literature with the documented adaptations can guide strategies aimed toward producing this reversal. The motivation underlying the interventions seen in the current literature originates from two complementary areas; one stems from aging and sports to target enhancement of performance while the second, based in tendinopathies, sets the goal of returning to prior activity after abolishing pain. Both research communities target the local tissue, despite the mechanical nature and demand of the first and the analgesic roots of the other.

It was established as far back as 2010 (Arya and Kulig, 2010; Child et al., 2010) that morphological changes within a tendon, attributed to degeneration, are accompanied by diminished intratendinous mechanical and material properties. These impaired tendon mechanics have given rise to intervention strategies, typically seen as a progressive resistance exercise program in the performance enhancement literature (Bohm et al., 2015). However, pathology carries with it three common issues that do not readily come to mind when the primary goal is limited to performance. The first issue relates to tendon morphology: although training-induced increases in Achilles tendon loading stimulate uniform increases in collagen production as seen by hypertrophy and improved elastic modulus (Bohm et al., 2015; Wiesinger et al., 2015; Magnusson and Kjaer, 2019), the pathological tendon’s thickening is non-homogenous (Maffulli et al., 2003; Cook and Purdam, 2008; Magnusson and Kjaer, 2019). The focal thickening is attributed to retention of water (Cook and Purdam, 2008; Magnusson and Kjaer, 2019) and thinner, disorganized collagen fibers (Maffulli et al., 2003), explaining the heightened mechanical compliance. This difference in morphological response begs the consideration that the pathological tendon may not respond to resistance exercise with the same efficiency as the healthy tendon.

The second issue is attributed to the time course of adaptations to tendon degeneration. We expect that the peripheral and central adaptations to a compliant tendon occurred over an extended time interval, and once established, are continuously reinforced by the persistence of said compliance. The late adaptations may require more response time under traditional treatment, reducing the efficiency of such protocols. The final matter is that presence of pain alters the body’s ability to undergo and respond to resistance exercise (Henriksen et al., 2011). Despite these challenges, tendon-targeted, slow progressive loading remains a viable strategy to initiate reversal of tendon alterations and the accompanying adaptations.

Previous reports indicate reduction of pain in as little as 2 or 3 weeks, suggesting that marked changes in the discussed alterations and adaptations will require longer periods of time (Murphy et al., 2018). On the other hand, the time-course of change in healthy tendon’s stiffness, due to resistance exercise, averages 12–14 weeks (Arampatzis et al., 2007, 2010; Kubo et al., 2010, 2012; Fouré et al., 2013; Wiesinger et al., 2015) with a suggested minimum of 8 weeks for a large effect (Bohm et al., 2015). Improvements in the elastic modulus show a similar trajectory. In contrast, alterations in cross-sectional area seem to require different exercise parameters. A meta-analysis on the effects of exercise on healthy tendons determined that protocols utilizing contractions lasting only one second at loads lower than 80% maximal voluntary contraction (MVC) were unable to induce improvements in cross-sectional area (Bohm et al., 2015). Furthermore, previous interventional studies on Achilles tendinopathy have reported the successful reduction of abnormal cross-sectional area via the use of heavy-resistance training (Magnusson and Kjaer, 2019). Therefore, our overall consensus is to aim for three main components in interventions for Achilles tendinopathy: (1) resistance exceeding body weight, (2) a high number of multi-second repetitions per day (up to 180 repetitions, depending on the stage of intervention), and (3) duration of the program in months as opposed to weeks. We propose that only a sufficient enhancement in tendon stiffness will result in reversibility of long-term neuromechanical adaptations. This statement is purely speculative in nature, largely due to the absence of studies monitoring intervention-based changes in tendon morphological, material, and mechanical properties in combination with the functional effects of these properties. We would like the readers to note that the above recommendations are merely meant to guide the development of new protocols rather than suggesting specific exercises.

As a means of supporting this proof of concept, we present data from a single subject with unilateral Achilles tendinosis who showed the same neuromechanical adaptations as the participants in Chang and Kulig,’s 2015 study (Chang and Kulig, 2015). The subject, an active male in his 40’s with two prior episodes of tendon pain (10 years and 1 year prior to the data collection, respectively), underwent a progressive-resistance, slow-rate exercise regimen based off of the Alfredson protocol (Alfredson et al., 1998). Prior episodes were managed by active rest. Soon after the onset of the current episode (which included perception of stiffness in the morning and pain during running), the athlete’s Achilles tendons were imaged and a significant focal thickness was discovered on the left side. After 2 weeks of active rest, when pain subsided, written informed consent was obtained and laboratory tests were commenced (approved by the IRB at the University of Southern California). Experimental data were collected in a motion analysis laboratory before and after the 12-week exercise regimen (Table 1). The parameters of interest are the same as those described in the “Multi-Level Physiological Adaptations” section. For a complete description of the laboratory methodology, please refer to the 2015 manuscript (Chang and Kulig, 2015). Furthermore, to bolster the interpretability of the intervention data, we compared the pre and post data to reliability values from an earlier repeated-measures experiment. The standard error of measure (SEM) values from this latter experiment are available in the 2015 manuscript referenced above.

**TABLE 1:** Morphological and neuromechanical variables before and after a 12-week progressive-resistance heel-lowering exercise intervention.

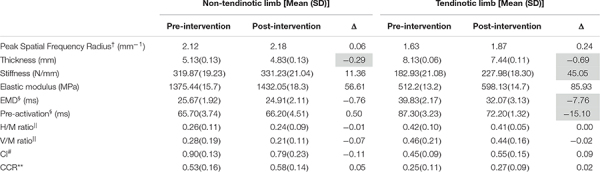

Single subject data are shown in two columns where “tendinotic” refers to the involved limb and “non-tendinotic” refers to the contralateral, non-involved limb. Delta (Δ) represents the difference between the pre- and post-intervention assessments. All data are reported as mean (standard deviation). Standard deviations for each variable were obtained from multiple measurements (3 to 5) on this subject. Shaded numbers indicate Δ exceeded the minimal detectable changes (MDC) for data from our lab. The MDC thresholds were calculated from standard error of measurement (SEM) values derived from reliability tests on five subjects. Please see Chang and Kulig (2015) for the relevant SEMs. ^†^Higher peak spatial frequency radius indicates less separation between collagen bundles (more bundles per mm); ^§^Electromechanical delay (EMD) and pre-activation represent the temporal efficiency of force transmission and the feedforward control response to that temporal efficiency, respectively; ^∥^H-reflex (H) and V-wave (V) ratios correspond to measures of spinal and supraspinal neural drive, respectively, and are normalized by the maximum M-wave (M) found during peripheral stimulation. ^#^Contribution index (CI) closer to 0 represents increased sagittal-plane synergist activity (peroneus longus) relative to the gastrocnemius and soleus. **Co-contraction ratio (CCR) closer to 0 indicates decreased antagonist activity (tibialis anterior) relative to the three plantar flexors used in the CI variable.

Taken together, the following information can be gathered from these preliminary data (Table 1). The focal thickness of the degenerated tendon diminished by 8% while the tendon stiffness, though still considerably lower than the non-involved side, increased by 24%. There was an observable EMD reduction of 19.5%, followed by delayed onset of gastrocnemius pre-activation (onset is closer to the time of ground contact by 17.3% of the pre-intervention time). In other words, there was improvement in the temporal efficiency of the mechanical system and the body’s feedforward control response was subsequently adjusted to match that temporal efficiency. Although these are notable improvements that exceed the minimal detectable change (MDC) for data collected in our laboratory, they did not entirely eradicate the between-limb differences in these parameters. Please see Chang and Kulig (2015) for the standard error of measurement values used to calculate the MDC. Moreover, changes in the reflex and feedback variables as well as task-specific muscle activation patterns did not exceed the MDC.

**FIGURE 1:**
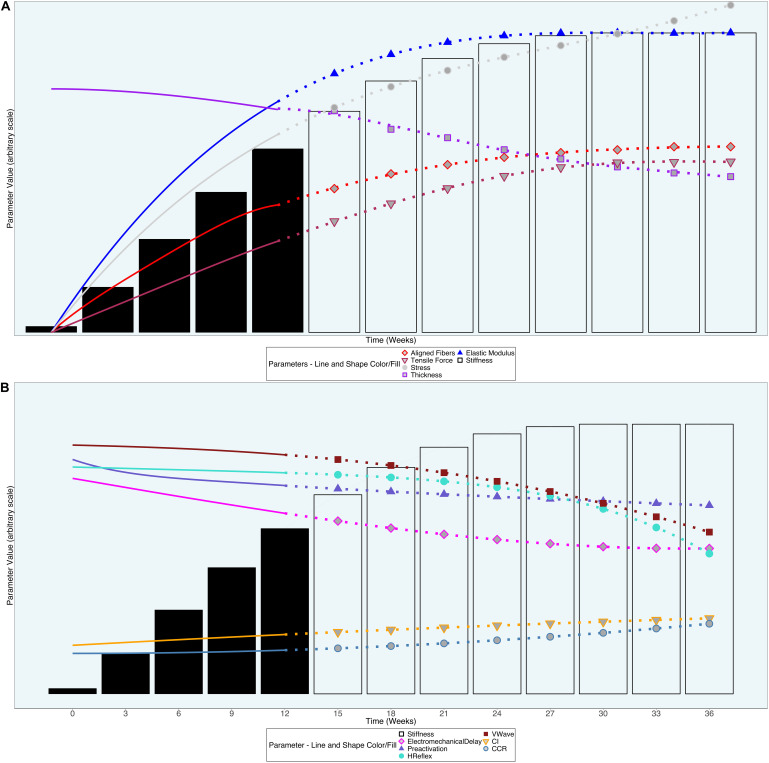
Hypothetical model of the time course of changes that may occur during progressive loading-based training of the degenerated Achilles tendon. **(A)** *Tendon morphological parameters*. Progressive increases in the external load (not depicted) will create concomitant increases in tensile force (red; filled diamonds) and therefore stress (gray; circles). This will in turn stimulate much-needed deposition of collagen but focal thickness reduction will likely outweigh the deposits, reducing the tendon’s overall thickness (purple; filled squares) while improving the ratio of aligned to non-aligned fibers (maroon; filled inverse triangles). The overall result is improvement in tendon elastic modulus (blue; triangles) and stiffness (black bars). **(B)** *Neuromechanical adaptations to tendon degeneration*. Once a sufficient improvement in tendon stiffness (black bars) is observed, electromechanical delay (magenta; filled diamonds) will decrease, allowing triceps surae pre-activation onset (purple; triangles) to occur closer to ground contact. Reduced duration of muscle activity may then impact alpha motor neuron excitability (H-reflex; turquoise, circles), descending cortical drive (V-wave; dark red; squares), and the simultaneous activity of multiple muscles (Contribution index and co-contraction ratio; orange and steel blue; filled inverse triangles and filled circles). The unfilled black bars, dotted lines, and symbols in both **(A)** and **(B)** indicate that the corresponding values are hypothetical and require laboratory testing for confirmation. Figure format informed by Wiesinger et al. (2015).

In Figure 1A we present a hypothetical 36-week timeline for the reversal of changes in tendon morphology and associated properties. For clarity, we present the parameters on an arbitrary scale. We acknowledge that the time course of changes will likely be non-linear, with greater response to the stimulus in the early portions of the intervention. However, we suggest that the progressive increase in stimulus, as well as the inherent ability of the degenerated tendon and the organism to continuously adapt, will prevent early cessation of the reversal.

We expect that the first changes will involve the intratendinous morphology. The first levels of external load should induce collagen repair and synthesis, thereby improving the percentage of aligned collagen fibers (Magnusson and Kjaer, 2019). One effect of this improvement in aligned fibers would be to reduce the ratio of proteoglycans to collagen, which should create an impetus for removal of the water in the tendon, ultimately reducing the focal thickness. However, the changes in thickness are thought to eventually plateau due to further increases in collagen offsetting the reduction in water content.

The increase in aligned collagen fiber percentage is hypothesized to begin the process of normalizing the modulus of elasticity and stiffness (Wiesinger et al., 2015). We suggest that increases in the modulus of elasticity indicate a greater number of properly aligned and healthy collagen fibers, thus representing a reduction in the ratio of fibers with permanent versus non-permanent damage. This underlying improvement in healthy collagen fiber numbers would in turn suggest a greater ability of the tendon to resist stress (deforming force normalized by area). On the other hand, fibers that have suffered permanent damage (plastic deformation) would undergo a larger amount of strain (elongation relative to original length) for a given amount of stress. The direct effect of healthy collagen fibers on the modulus of elasticity indicates that improvement in the modulus may occur prior to and may facilitate recovery of tendon stiffness, a notion also suggested by Bohm et al. (2015).

In Figure 1B, we present the additional speculative reversibility of supraspinal and spinal adaptations. Once the tendon stiffness increases, the temporal efficiency of force transmission (EMD) should improve, allowing a subsequent delay in the pre-activation onset, thus approximating pre-pathology timing. In theory, the delayed pre-activation would lead the nervous system to decrease the excitability of the alpha motor neuron, thus removing the need for the compensatory heightened H-reflex and V-wave. The co-contraction ratio (CCR) and contribution index (CI) may not de-adapt until there is a sufficient reduction in pain and other symptoms, permitting full utilization of the triceps surae and the opposing dorsiflexors. However, due to the failure of these last four parameters to reach MDC in our lone participant, we currently lack sufficient information to delineate the order and timing of their normalization.

## Eliminating the Root Cause – Where Do We Start?

These data from a single subject illustrate that reversing the multi-level adaptations in the motor control system is a reachable goal. However, the current treatment strategy focusing on pain reduction and returning to activity appears insufficient. This is largely a result of the lengthy duration, which would require numerous clinical visits as well as extensive time commitment at home, both of which may affect adherence. To achieve reversal of multi-level adaptations accompanying Achilles tendinosis, the initial stages of rehabilitation should target the morphology and associated mechanical (structural) and material deficiencies of the tendon. The adaptations remaining after this initial stage of mechanical recovery are closely aligned with variables seen in the motor control and motor learning field. This indicates that motor learning strategies aiming to undo adaptation at the higher levels of the control system are good candidates for incorporation into the treatment regimen. Therefore, we conclude this perspective with the assertion that the combination of progressive resistance exercise, motor learning, and currently unknown exercises should prove suitable for reversing the effects of Achilles tendon degeneration and improving the quality of life of those affected.

## Data Availability Statement

The datasets generated for this study are available on request to the corresponding author.

## Ethics Statement

The studies involving human participants were reviewed and approved by the University of Southern California, IRB. The patients/participants provided their written informed consent to participate in this study.

## Author Contributions

KK and Y-JC conceived and designed the study. Y-JC acquired and analyzed the data. KK and Y-JC interpreted the data. KK, Y-JC, and DO-W drafted, revised, and approved the final version of the manuscript.

## Conflict of Interest

The authors declare that the research was conducted in the absence of any commercial or financial relationships that could be construed as a potential conflict of interest.
